# The efficacy of first-line tyrosine kinase inhibitors combined with co-medications in Asian patients with EGFR mutation non-small cell lung cancer

**DOI:** 10.1038/s41598-020-71583-w

**Published:** 2020-09-11

**Authors:** Vincent Yi-Fong Su, Kuang-Yao Yang, Ting-Yu Huang, Chia-Chen Hsu, Yuh-Min Chen, Jiin-Cherng Yen, Yueh-Ching Chou, Yuh-Lih Chang, Chien-Hui He

**Affiliations:** 1 Department of Internal Medicine, Taipei City Hospital, Taipei, Taiwan; 2 Department of Pharmacy, Taipei Veterans General Hospital, Taipei, Taiwan; 3 Department of Chest Medicine, Taipei Veterans General Hospital, Taipei, Taiwan; 4 Department and Institute of Pharmacology, National Yang-Ming University, Taipei, Taiwan; 5 Faculty of Medicine, School of Medicine, National Yang-Ming University, Taipei, Taiwan; 6 School of Pharmacy, Taipei Medical University, Taipei, Taiwan; 7 School of Medicine, Taipei Medical University, Taipei, Taiwan; 8 Faculty of Pharmacy, National Yang-Ming University, Taipei, Taiwan; 9 Cancer Progression Research Center, National Yang-Ming University, Taipei, Taiwan

**Keywords:** Cancer, Medical research, Oncology

## Abstract

The real-world efficacy of epidermal growth factor receptor (EGFR)-tyrosine kinase inhibitors (TKIs) in patients with advanced non-small cell lung cancer (NSCLC) harboring EGFR-activating mutations remains unclear. We conducted a retrospective cohort study using data from the claims database of Taipei Veterans General Hospital to perform direct comparisons of these three EGFR-TKIs (gefitinib, erlotinib, and afatinib) combined with co-medications (metformin, statins, antacids, and steroids). Stage IIIB and IV NSCLC patients with EGFR mutations receiving EGFR-TKIs as first-line treatment for > 3 months between 2011 and 2016 were enrolled. The primary endpoint was time to treatment failure (TTF). Patients who had received co-medications (≥ 28 defined daily doses) in the first 3 months of EGFR-TKI therapy were assigned to co-medications groups. A total of 853 patients treated with gefitinib (n = 534), erlotinib (n = 220), and afatinib (n = 99) were enrolled. The median duration of TTF was 11.5 months in the gefitinib arm, 11.7 months in the erlotinib arm, and 16.1 months in the afatinib arm (log-rank test, P < 0.001). After adjustments, afatinib showed lower risk of treatment failure compared with gefitinib (hazard ratio [HR] 0.54, 95% confidence interval [CI] 0.41–0.71) and erlotinib (HR 0.62, 95% CI 0.46–0.83). The risk of treatment failure in patients treated with EGFR-TKIs who received concomitant systemic glucocorticoid therapy was higher than in those treated with EGFR-TKI monotherapy (HR 1.47, 95% CI 1.08–2.01). Afatinib or erlotinib use was associated with a lower risk of treatment failure in patients with advanced NSCLC harboring EGFR mutations compared to gefitinib use. Concurrent use of systemic glucocorticoids was linked to higher risk of treatment failure.

## Introduction

Lung cancer is one of the leading causes of cancer death, with limited treatment options available for advanced-stage disease^1^. Non-small cell lung cancer (NSCLC) is the most common type of lung cancer, accounting for approximately 85% of primary lung cancers. In addition, most patients with NSCLC present with advanced or metastatic disease at the time of diagnosis. Recent research aiming at the treatment of NSCLC has been focused on the inhibition of epidermal growth factor receptor (EGFR) mutations through the administration of EGFR-tyrosine kinase inhibitors (TKIs). In terms of geographical distribution, the Asia–Pacific subgroup exhibited the higher frequency of EGFR mutation in patients with NSCLC (47%)^2^. Within this region, the highest and lowest frequencies of EGFR mutation in patients with NSCLC were observed in Taiwan (57%) and Singapore (40%), respectively. In NSCLC, > 90% of the known EGFR-activating mutations are exon 19 deletions and Leu858Arg mutations (L858R)^3^. EGFR mutations may result in constitutive activation of EGFR-tyrosine kinases, leading to cell proliferation or anti-apoptosis, regardless of the presence of extracellular ligands.

EGFR-TKIs have become the cornerstone therapy for patients with NSCLC harboring EGFR mutations, and are recommended as first-line treatment for patients with advanced NSCLC positive for EGFR mutations^4^. Three oral EGFR-TKIs, namely gefitinib (Iressa), erlotinib (Tarceva), and afatinib (Giotrif), have recently been approved for first-line use in advanced patients with NSCLC harboring EGFR mutations. Gefitinib and erlotinib are first-generation EGFR-TKIs that reversibly inhibit EGFR. In contrast, afatinib is an irreversible EGFR-TKI, considered as a second-generation EGFR-TKI^4^. These three TKIs have shown improved response rates and progression-free survival (PFS) compared to first-line conventional platinum-based chemotherapy^4–7^. A recent phase III, randomized, controlled trial^8^, (LUX-Lung 7), revealed that afatinib exerted significant benefits in PFS compared to gefitinib in NSCLC patients with EGFR-activating mutations (hazard ration [HR] 0.73; 95% confidence interval [CI] 0.57–0.95). However, phase III, randomized, controlled trials mainly enroll lung cancer patients with a good performance. Therefore, the effectiveness of TKI use in NSCLC patients presenting with impaired performance status (PS) in real-world clinical practice remains unclear. Other drugs that affect metabolism, such as statins^9^ and metformin^10^, have also been reported to have antitumor effects on NSCLC. Previous reports^11,12^ showed that antacids and corticosteroids use may compromise the clinical efficacy of EGFR-TKIs in advanced NSCLC patients. However, the real-world effects of co-medications use in patients with NSCLC receiving EGFR-TKI therapy remains unclear. The objective of the present study was to analyze the distinct effects of the three TKIs and co-medications use in NSCLC patients with EGFR mutations in a real-world setting.

## Methods

### Data source

This study was conducted at Taipei Veterans General Hospital, a 3,000-bed tertiary referral hospital in Taiwan, treating approximately 800 newly-diagnosed cases of lung cancer per year. The stage of NSCLC was determined according to the 7th edition of the American Joint Committee on Cancer (AJCC) TNM staging system. Classical EGFR mutations were exon 19 deletion and point mutation of exon 21 (L858R). Other EGFR mutations included any other mutations of exon 18, 19, and 21. All three EGFR-TKIs have been reimbursed by the Taiwan National Health Insurance (NHI) program for the first-line treatment of patients with advanced NSCLC (stage IIIB or IV) with EGFR-activating mutations. Gefitinib, erlotinib, and afatinib have been used as first-line therapy since June 2011, November 2013, and May 2014, respectively. All methods were performed in accordance with the Declaration of Helsinki. Informed consent was waived because of the retrospective design and the analysis used anonymous clinical data. The present study was approved by the Institutional Review Board of Taipei Veterans General Hospital (VGHIRB NO: 2018-06-013CC).

The information of NSCLC patients treated with EGFR-TKIs reimbursed by the NHI program was externally reviewed by the NHI organization every 3 months. Chest computed tomography (CT) combined with other medical imaging modalities (i.e., brain magnetic resonance imaging, whole body bone scan, or positron emission tomography–CT) were used to re-evaluate the disease status. The status of NSCLC was considered under control (e.g. complete response, partial response, or stable disease) according to the New Response Evaluation Criteria in Solid Tumors (RECIST) guidelines^13^. Following the re-evaluation, EGFR-TKIs could have been prescribed and reimbursed again by the Taiwan NHI program.

### Study design and population

We conducted a retrospective analysis to evaluate the effects of three EGFR-TKIs as first-line treatment in patients with advanced NSCLC harboring EGFR mutations from January 2011 to December 2016. All patients underwent treatment at the Taipei Veterans General Hospital. Patient inclusion criteria were: (I) new or recurrent stage IIIB/IV NSCLC; (II) presence of somatic EGFR mutations; and (III) treated with EGFR-TKIs as first-line therapy for ≥ 3 months. Patient exclusion criteria were: (I) age < 20 years; (II) diagnosis of other cancer prior to lung cancer diagnosis or within 1 year after lung cancer diagnosis; and (III) negative or lack of EGFR mutation analysis. All patients were followed up until the date of death, or 31 December 2017, or the end of the 2-year follow-up period, whichever occurred first.

### Potential confounders and classification of severity

In the analysis of the effects of EGFR-TKI administration on disease control, age, sex, smoking status, AJCC TNM stage, EGFR mutation types, baseline brain metastases (BM), Eastern Cooperative Oncology Group PS (ECOG PS) score at the time of lung cancer diagnosis, initial radiotherapy (RT), DCCI, and co-medications were considered to be potential confounders.

Baseline BM was used to identify patients with BM prior to the administration of EGFR-TKIs or within 1 month after receiving EGFR-TKI treatment. Initial RT meant that patients received RT within 3 months after the administration of EGFR-TKIs. Comorbidities were defined as those diagnosed in patients prior to the initiation of EGFR-TKI therapy.

### Effect of exposure to co-medications

Information on co-medications was extracted to analyze the effects of EGFR-TKI therapy on disease progression and mortality. The four groups of co-medications were: glucocorticoids (betamethasone, dexamethasone, cortisone, prednisolone, hydrocortisone, triamcinolone, and methylprednisolone); antacids (proton pump inhibitors: omeprazole, esomeprazole, lansoprazole, rabeprazole, dexlansoprazole, and pantoprazole; histamine-2-receptor antagonists: cimetidine, ranitidine, famotidine, nizatidine, and roxatidine); statins (simvastatin, pravastatin, fluvastatin, atorvastatin, rosuvastatin, and pitavastatin); and metformin. Patients prescribed ≥ 28 cumulative defined daily doses (cDDDs) of each group of co-medication within the first 3 months of receiving EGFR-TKI therapy were assigned to respective co-medication groups. The DDD of each co-medication was estimated in accordance with 2017 ATC/DDD WHO Index^14^.

### The accuracy of the diagnosis of NSCLC and the EGFR mutation status

In this study, NSCLC was pathologically confirmed by a bronchoscopic- or computed tomography (CT)-guided biopsy, and/or surgical resections. The EGFR mutation status of tumors was determined through analysis of DNA extracted from formalin-fixed, paraffin-embedded, archival tumor tissues. EGFR mutation analysis was performed using polymerase chain reaction-direct sequencing and scorpion/amplified refractory mutation system technology. The diagnosis of NSCLC in this study was externally reviewed by the NHI organization. Moreover, the EGFR mutation status must be reviewed by the NHI organization. Therefore, in this study, the diagnosis of NSCLC and the EGFR mutation status was reliable and exhaustive.

### Outcomes and survival analysis

The primary endpoint was time to treatment failure (TTF). TTF was defined as the time from the first day of EGFR-TKI administration to the date of treatment failure. In this study, treatment failure was defined as the date of EGFR-TKI discontinuation, judged by the treating physicians. The secondary endpoints were overall survival (OS) and BM-free survival. OS was defined as the time from the first day of EGFR-TKI treatment initiation to all-cause death or lost to follow-up, or the end of the follow-up period. BM-free survival was defined as the time from the first day of EGFR-TKI treatment initiation to the day of detection of new BM in NSCLC patients without baseline BM at the time of enrollment.

### Statistical analyses

All data are expressed as mean ± standard deviation (SD) or percentage (%), unless otherwise stated. Differences in continuous variables between the three EGFR-TKI groups were compared using analysis of variance. Differences in categorical variables were compared using Pearson’s χ^2^ test. Survival was assessed using the Kaplan–Meier curves, and the log-rank test was used to compare differences between groups. The HR and 95% CI of the risk of treatment failure and mortality and new BM among different EGFR-TKI groups were calculated using the Cox proportional hazards regression analysis. The factors used as covariates in the multivariate Cox proportional hazards model are shown in Supplementary Table 1. The propensity score was used as a covariate in the regression model in the sensitivity analysis. All factors with P < 0.1 in the univariate analyses were included in the Cox multivariate analysis. Accompanying 95% CIs were calculated after adjustment for possible confounding factors. Two-tailed P values < 0.05 were considered statistically significant. All analyses were performed using the SAS statistical software version 9.4 (Cary, NC, USA). Two-sided P < 0.05 were considered statistically significant.

## Results

### Clinical characteristics of the study population

We identified 1,285 patients with NSCLC diagnosed between 1 January 1997 and 31 December 2012, who received EGFR-TKIs as first-line treatment for ≥ 3 months. Patients with other types of cancer prior to or within 1 year after the diagnosis of NSCLC (n = 278), those aged < 20 years, and those with negative or lack of EGFR mutations analysis (n = 154), were excluded from the analysis. The final study population consisted of 853 NSCLC patients, who received first-line treatment with gefitinib (n = 534), erlotinib (n = 220), or afatinib (n = 99) (Supplementary Fig. 1). The baseline characteristics of the patients are shown in Supplementary Table 1.

Females were more predominant (63.7%) and most patients had stage IV disease (96.1%) and adenocarcinoma (94.1%). Among the study groups, patients receiving gefitinib (66.8 years) were significantly older than those treated with erlotinib (63.0 years) or afatinib (64.1 years). There were no differences in the stage of disease, pathology report, initial RT, and DCCI between the groups. The gefitinib group had a higher prevalence of females and an ECOG PS score ≥ 2 compared to the other groups. The erlotinib group had a higher prevalence of L858R mutation, baseline BM, and initial RT compared to the other groups. The afatinib group had a higher prevalence of smokers and exon 19 deletion compared to the other groups.

The co-medications prescribed to the three groups are shown in Supplementary Table 1. Of the 853 patients, 40 (4.7%), 38 (4.5%), 92 (10.8%), and 53 (6.2%) patients received metformin, statins, antacids, and systemic glucocorticoids, respectively, during the first 3 months of EGFR-TKI administration. The gefitinib group included more patients co-treated with antacids (12.9%) than the erlotinib (6.8%) and afatinib (8.1%) groups (P = 0.0320).

### Effect of EGFR-TKIs on treatment failure in NSCLC patients with EGFR-activating mutations

**Figure 1:**
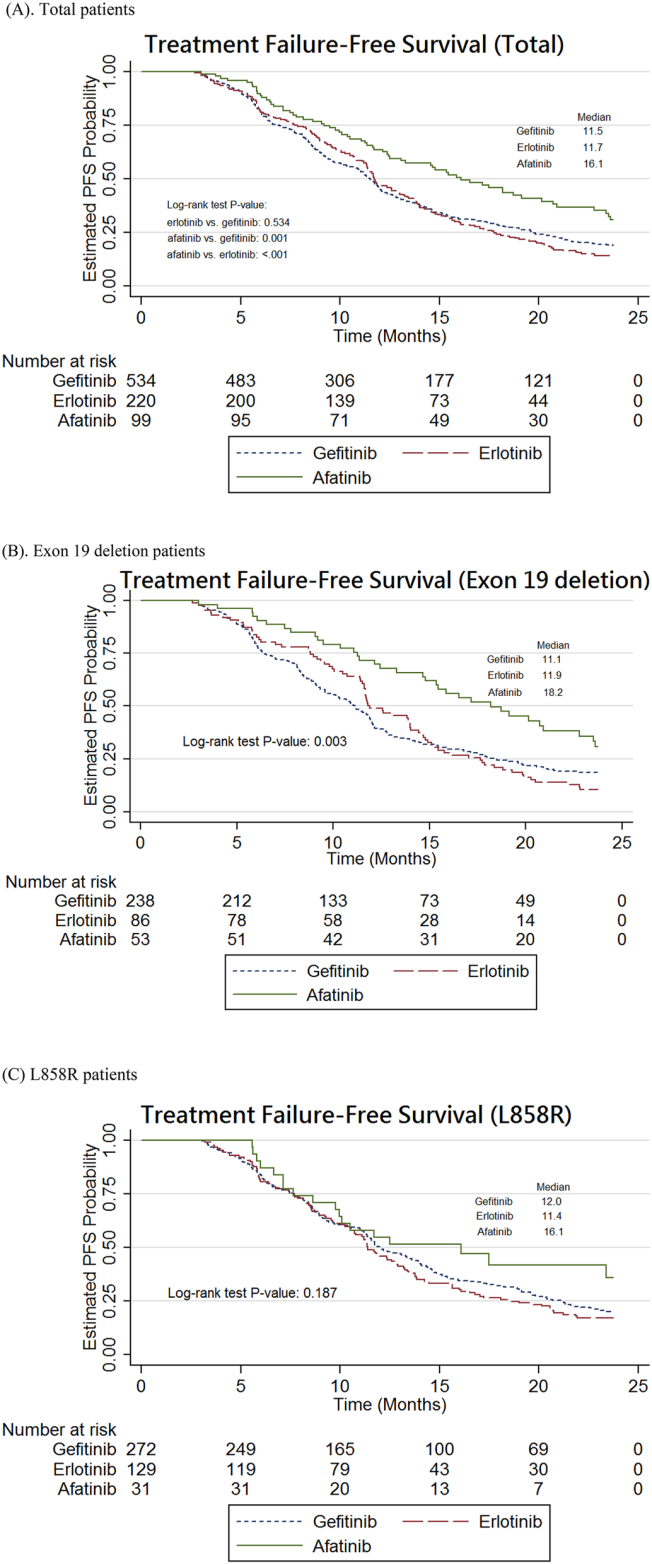
Kaplan–Meier curve: treatment failure-free survival in advanced NSCLC patients with EGFR mutations. (**A**) Total patients. (**B**) Exon 19 deletion patients. (**C**) L858R patients.

Other EGFR mutations (HR 1.68, 95% CI 1.18–2.39, P = 0.004), and ECOG PS ≥ 2 (HR 1.31, 95% CI 1.06–1.62, P = 0.012), and baseline BM (HR 1.64, 95% CI 1.38–1.94, P < 0.001) were identified as independent risk factors of treatment failure in NSCLC patients with EGFR-activating mutations who received EGFR-TKIs as first-line treatment. In contrast, age ≥ 65 years (HR 0.78, 95% CI 0.67–0.92, P = 0.002) and female sex (HR 0.75, 95% CI 0.63–0.91, P = 0.003) were associated with significantly lower risks of treatment failure (Table 1). Using the Kaplan–Meier analysis, it was shown that TTF was significantly longer in the afatinib group than in the other two groups (afatinib vs. erlotinib, log-rank test, P < 0.001; afatinib vs. gefitinib, log-rank test, P = 0.001; Fig. 1A). The median TTF was 11.5, 11.7, and 16.1 months for gefitinib, erlotinib, and afatinib, respectively. Importantly, the median TTF in NSCLC patients with the exon 19 deletion was much longer in the afatinib group compared with that observed in the other groups (i.e., gefitinib 11.1 months vs. erlotinib 11.9 months vs. afatinib 18.2 months; log-rank test, P = 0.003; Fig. 1B). In contrast, there were no differences observed in median TTF in NSCLC patients with the L858R mutation between the groups (i.e., gefitinib 12.0 months vs. erlotinib 11.4 months vs. afatinib 16.1 months; log-rank test, P = 0.187; Fig. 1C).

**Table 1:** Risk factor analyses of treatment failure and all-cause mortality in patients with stage IIIB or IV NSCLC harboring EGFR-activating mutations.

	Treatment failure^a^		All-cause mortality^a^	
	HR (95% CI)	P-value	HR (95% CI)	P-value
Age ≥ 65	0.78 (0.67–0.92)	0.002	0.92 (0.75–1.12)	0.392
Female	0.75 (0.63–0.91)	0.003	0.72 (0.57–0.91)	0.005
**TKIs (Ref: Gefitinib)**				
Erlotinib	0.88 (0.73–1.05)	0.152	0.66 (0.52–0.85)	0.001
Afatinib	0.54 (0.41–0.71)	< 0.001	0.67 (0.45–1.00)	0.051
**TKIs (Ref: Erlotinib)**				
Gefitinib	1.14 (0.95–1.37)	0.152	1.51 (1.17–1.93)	0.001
Afatinib	0.62 (0.46–0.83)	0.001	1.01 (0.65–1.57)	0.961
**EGFR mutation (ref = Ex19d)**				
L858R	0.94 (0.80–1.10)	0.413	1.11 (0.91–1.35)	0.305
Other	1.68 (1.18–2.39)	0.004	1.31 (0.85–2.02)	0.228
Stage IIIB (ref = IV)	1.05 (0.69–1.60)	0.819	1.56 (0.95–2.58)	0.082
ECOG PS ≥ 2	1.31 (1.06–1.62)	0.012	2.26 (1.79–2.86)	< 0.001
Current or ever smoking	0.94 (0.76–1.16)	0.581	0.76 (0.58–1.00)	0.049
Receiving Radiotherapy	0.87 (0.70–1.09)	0.239	0.92 (0.70–1.19)	0.514
Baseline brain metastases	1.64 (1.38–1.94)	< 0.001	1.79 (1.45–2.22)	< 0.001
DCCI Score > 2 (Ref: 2)	0.86 (0.73–1.02)	0.077	1.06 (0.87–1.29)	0.584
**Co-medications**				
Metformin	1.09 (0.75–1.59)	0.657	0.82 (0.52–1.31)	0.411
Statins	1.06 (0.73–1.53)	0.774	0.93 (0.58–1.49)	0.768
Antacids	0.89 (0.69–1.15)	0.374	1.01 (0.75–1.36)	0.952
Glucocorticoids	1.47 (1.08–2.01)	0.015	1.85 (1.29–2.66)	< 0.001

^a^All factors were included in the Cox multivariate analysis.

**Figure 2:**
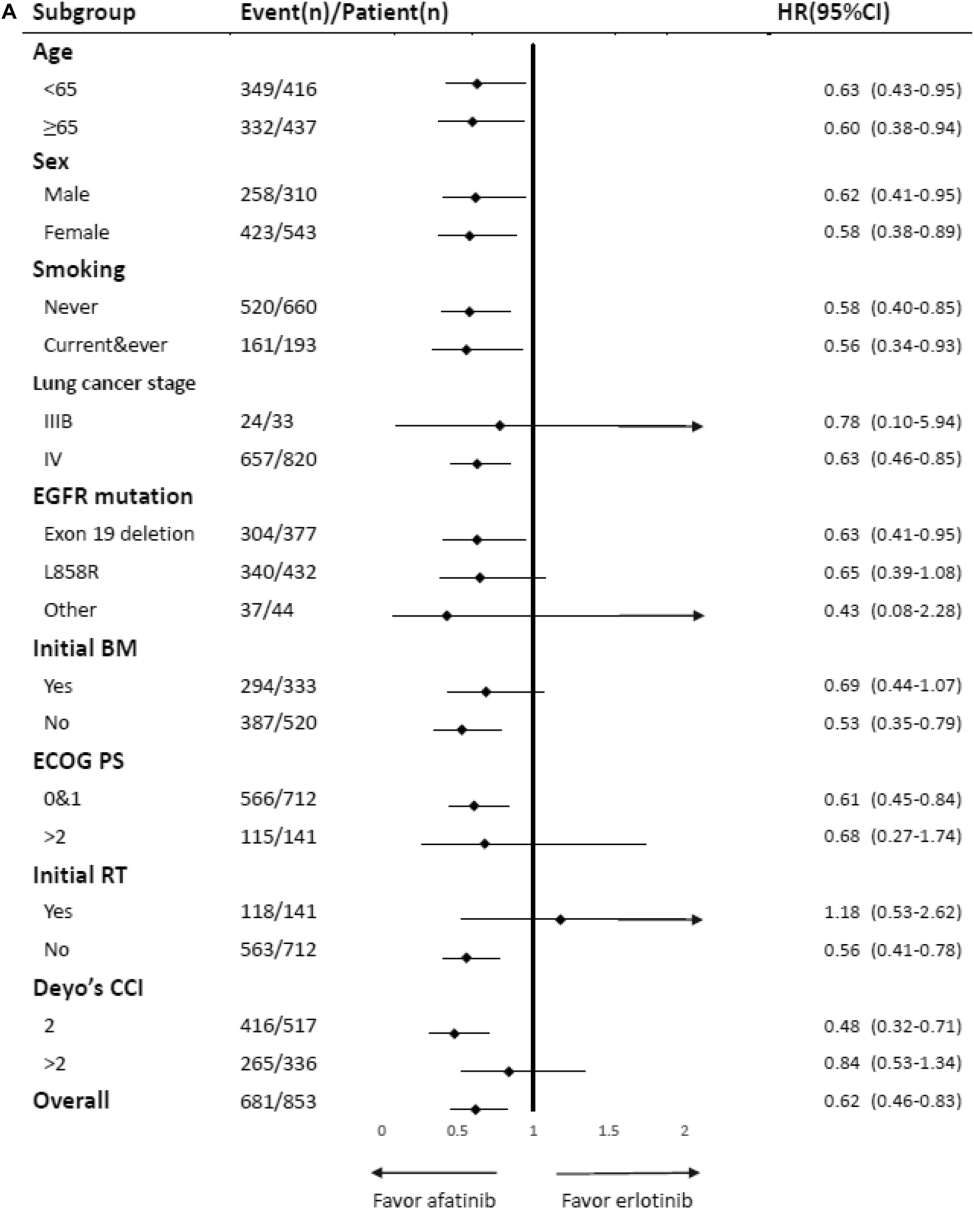

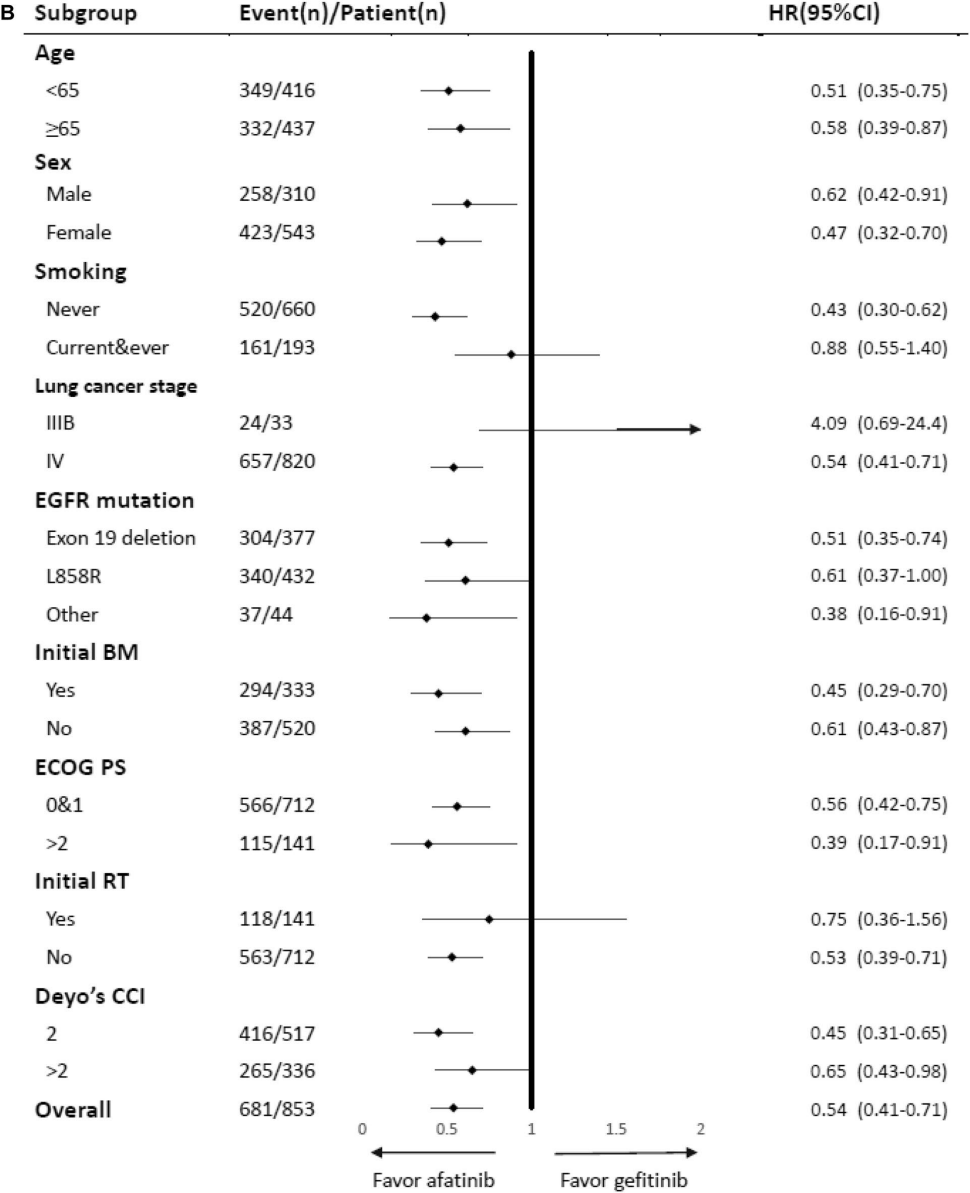

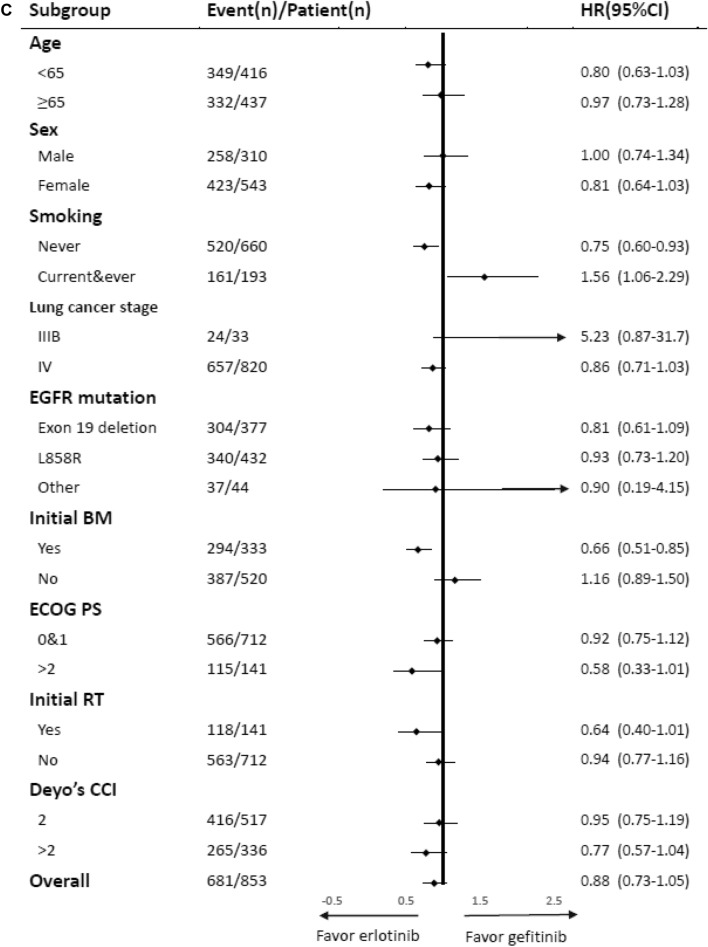

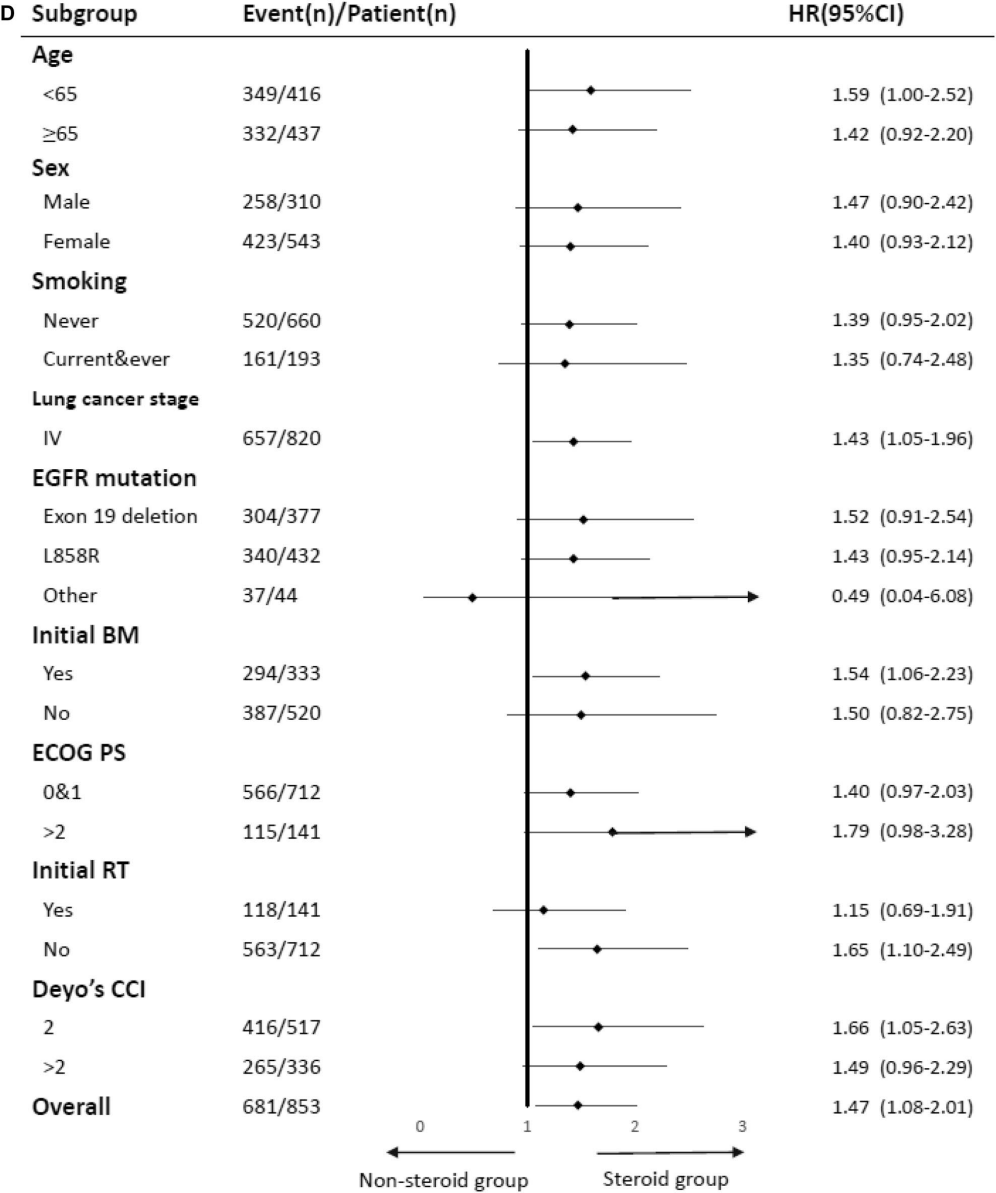
(**A**) Subgroup analysis for treatment failure: Comparison between afatinib and erlotinib. (**B**) Subgroup analysis for treatment failure: comparison between afatinib and gefitinib. (**C**) Subgroup analysis for treatment failure: Comparison between erlotinib and gefitinib. (**D**) Subgroup analysis for treatment failure: combination of glucocorticoids and TKIs.

After adjustment, afatinib showed lower risk of treatment failure compared with gefitinib (HR 0.54, 95% CI 0.41–0.71, P < 0.001) and erlotinib (HR 0.62, 95% CI 0.46–0.83, P = 0.001) in NSCLC patients with EGFR-activating mutations. However, there was no difference observed in risk of treatment failure between gefitinib and erlotinib in NSCLC patients with EGFR-activating mutations (Table 1). The results of subgroup analyses are presented in Fig. 2A–C. The HRs for the use of afatinib were significantly lower than those calculated for the other EGFR-TKIs in most subgroups, suggesting an independent role of afatinib in treatment failure.

### Effect of EGFR-TKIs on all-cause mortality and new BM in NSCLC patients with EGFR-activating mutations

**Figure 3:**
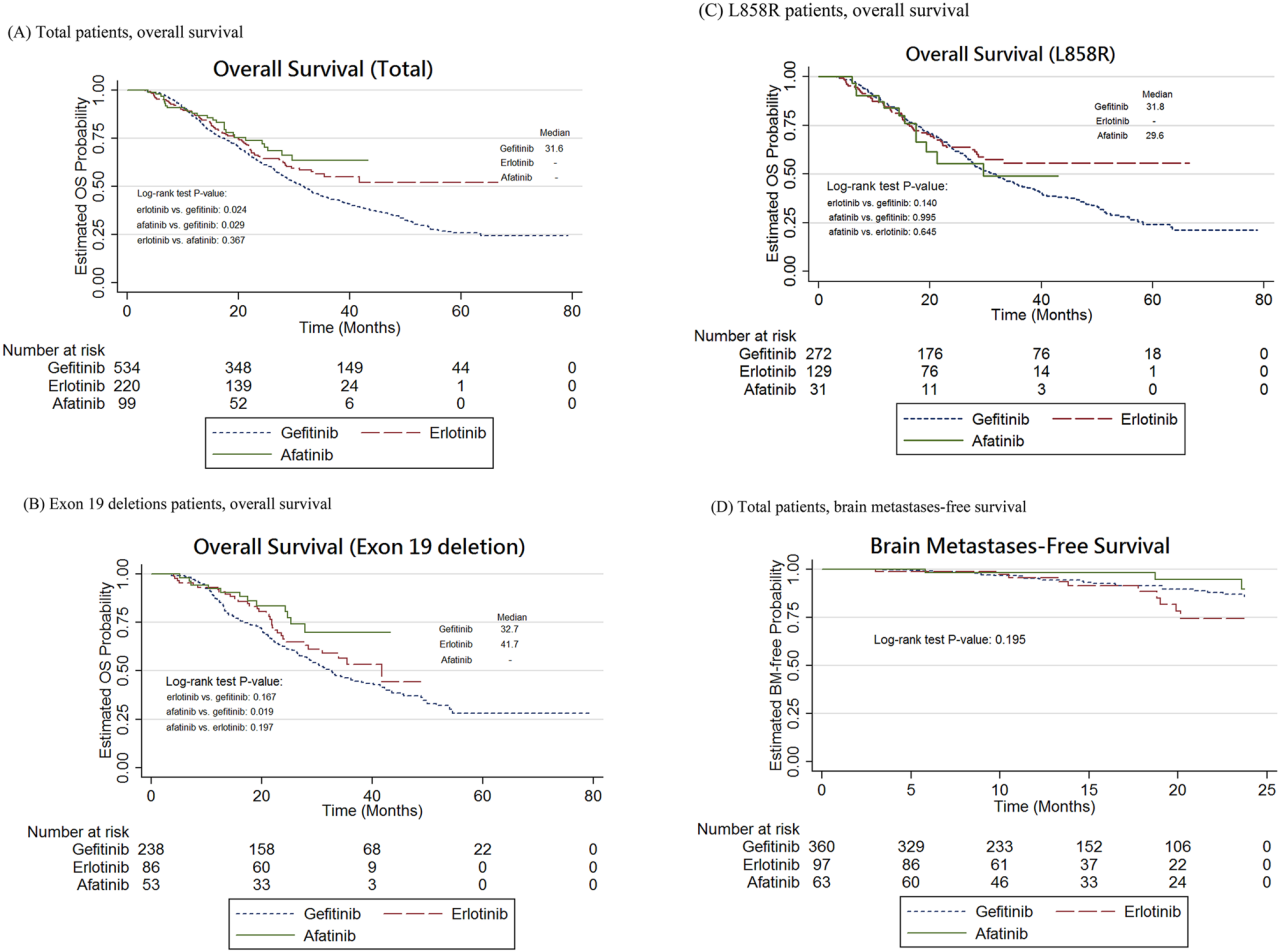
Kaplan–Meier curve: overall survival or brain metastases-free survival in advanced NSCLC patients with EGFR mutations. (**A**) Total patients, overall survival. (**B**) Exon 19 deletions patients, overall survival. (**C**) L858R patients, overall survival. (**D**) Total patients, brain metastases-free survival.

Using the Kaplan–Meier analysis, it was shown that the OS time was significantly longer in the afatinib and erlotinib groups than in the gefitinib group (afatinib vs. gefitinib, log-rank test, P = 0.029; erlotinib vs. gefitinib, log-rank test, P = 0.024; Fig. 3A). Importantly, OS in the afatinib group was significantly longer in NSCLC patients with the exon 19 deletion (P = 0.019), but not in those with the L858R mutation (Fig. 3B–C). However, there was no difference observed in BM-free survival between the groups (Fig. 3D). Moreover, after adjustment, no significant difference was observed in new BM between the groups (Supplementary Table 2).

### Effect of co-medications

**Figure 4:**
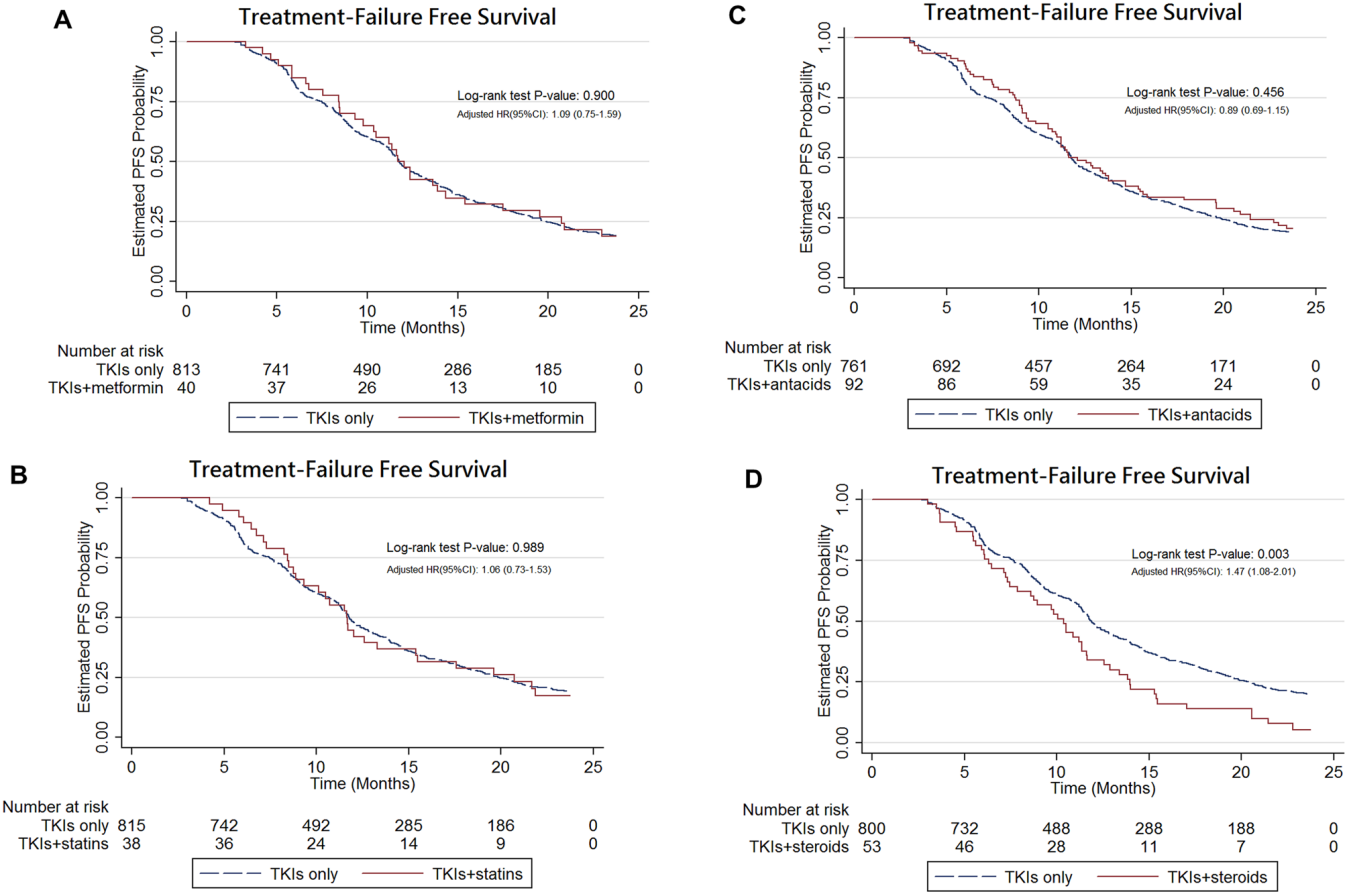
(**A**) Kaplan–Meier curve: treatment failure-free survival of advanced NSCLC patients with EGFR mutations treated with combinations of metformin and TKIs. (**B**) Kaplan–Meier curve: progression-free survival of advanced NSCLC patients with EGFR mutations treated with combinations of statins and TKIs. (**C**) Kaplan–Meier curve: progression-free survival of advanced NSCLC patients with EGFR mutations treated with combinations of antacids and TKIs. (**D**) Kaplan–Meier curve: progression-free survival of advanced NSCLC patients with EGFR mutations treated with combinations of glucocorticosteroids and TKIs versus TKI monotherapy.

**Figure 5:**
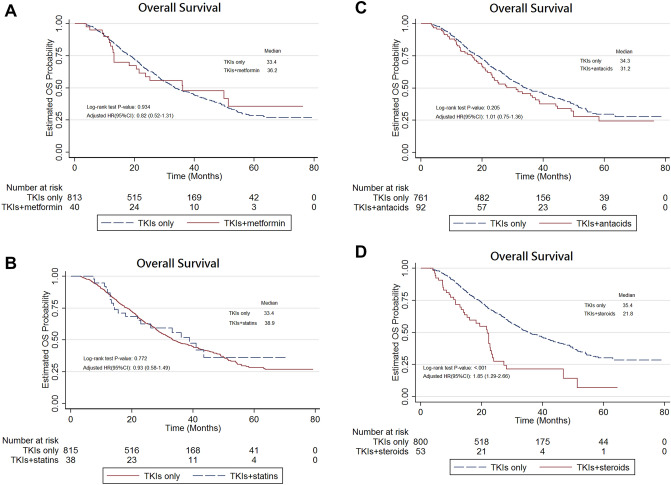
(**A**) Kaplan–Meier curve: overall survival of advanced NSCLC patients with EGFR mutations treated with combination of metformin and TKIs versus TKI monotherapy. (**B**) Kaplan–Meier curve: overall survival of advanced NSCLC patients with EGFR mutations treated with combination of statins and TKIs versus TKI monotherapy. (**C**) Kaplan–Meier curve: overall survival of advanced NSCLC patients with EGFR mutations treated with combination of antacids and TKIs versus TKI monotherapy. (**D**) Kaplan–Meier curve: overall survival of advanced NSCLC patients with EGFR mutations treated with combination of glucocorticoids and TKIs versus TKI monotherapy.

The Kaplan–Meier analysis showed that there were no significant differences in TTF (Fig. 4A–C) and OS (Fig. 5A–C) between those who received combination of EGFR-TKIs with three types co-medications (metformin, statins, or antacids) and those treated with EGFR-TKIs only. Importantly, EGFR-TKIs in combination with systemic glucocorticoids was associated with a significantly shorter TTF (log-rank test, P = 0.003; Fig. 4D) and OS (log-rank test, P < 0.001; Fig. 5D) compared to those observed following treatment with EGFR-TKIs only. After adjustment, the use of systemic glucocorticoids was associated with a significantly higher risk of treatment failure (HR 1.47, 95% CI 1.08–2.01, P = 0.015) and all-cause mortality (HR 1.85, 95% CI 1.29–2.66, P < 0.001; Table 1). The results of the subgroup analysis assessing the effect of systemic steroids on treatment failure are presented in Fig. 2D. The HRs for systemic glucocorticoids were significantly higher in certain subgroups, such as patients with age < 65 years, stage IV NSCLC, positive baseline BM, no initial RT, and DCCI = 2. The results of the sensitivity analyses adjusting for propensity scores on treatment failure are presented in Supplementary Table 3. After adjustment, afatinib showed lower risk of treatment failure compared with gefitinib (HR 0.56, 95% CI 0.42–0.73, P < 0.001) and erlotinib (HR 0.56, 95% CI 0.41–0.77, P < 0.001) in NSCLC patients with EGFR-activating mutations. The results of adjustment with propensity scores were similar to the results of adjustment without propensity scores (Table 1).

## Discussion

This study closely reflects real clinical practice. Our results revealed that the administration of afatinib was associated with significantly longer TTF versus erlotinib or gefitinib in NSCLC patients with EGFR-activating mutations who received EGFR-TKIs as first-line therapy. EGFR-TKIs combined with systemic glucocorticoids were associated with a significantly shorter TTF and OS compared to EGFR-TKIs without systemic glucocorticoids.

The LUX-Lung 7 trial^8^ reported statistically significant benefits in progression-free survival (PFS) in patients treated with afatinib compared to those observed following treatment with gefitinib as first-line therapy in NSCLC patients harboring EGFR-activating mutations (HR 0.73). The results of the present study are consistent with those of the LUX-Lung 7 trial. The administration of afatinib was associated with prolonged TTF versus gefitinib (HR 0.54; 95% CI 0.41–0.71, P < 0.001). In addition, use of afatinib was associated with prolonged TTF in NSCLC patients with exon 19 deletion versus those with L858R (median PFS: 18.2 months vs. 16.1 months, respectively). This finding was also consistent with the results of the LUX-Lung 3^6^, LUX-Lung 6^7^, and LUX-Lung 7^8^ trials. In this study, the TTF in the afatinib group was significantly longer than that calculated for the gefitinib group in most subgroup analyses after adjustments for all covariates. This finding is consistent with the findings of the LUX-Lung 7 trial, indicating the superiority of afatinib over gefitinib^8^.

Erlotinib has not been compared with afatinib in randomized controlled trials. To the best of our knowledge, this was the first study showing that use of afatinib was associated with prolonged TTF compared with that observed following the use of erlotinib. After adjustment, afatinib showed lower risk of treatment failure compared with erlotinib (HR 0.62; 95% CI 0.46–0.83, P = 0.001) in most subgroup analyses. A Taiwanese retrospective cohort study^15^ involving 448 patients with advanced NSCLC and EGFR-activating mutations who received EGFR-TKIs as first-line therapy showed that afatinib and erlotinib exerted significant benefits in PFS compared to gefitinib. As expected, in this study, the administration of afatinib and erlotinib was associated with prolonged TTF and OS compared with those observed following the administration of gefitinib. However, there was no difference observed in BM-free survival between the three EGFR-TKI groups.

Emerging evidence indicates the potential role of metformin^16^ and statins^17^ in decreasing the risk of cancer occurrence in humans. Observational data suggest that the use of metformin^10,18^ and statins^9^ may decrease the incidence of lung cancer and mortality. Mevalonate is the product of HMG-CoA via HMG-CoA reductase. Mevalonate metabolites have been shown to play a role in EGFR signaling pathway. There is some suggestion that HMG-CoA reductase inhibitors (statins) and EGFR-TKIs may act cooperatively to target EGFR-mediated signaling^19^. Metformin use may overcome the acquired resistance to EGFR-TKI in NSCLC. Metformin is able to synergize with other treatment in NSCLC including targeted therapy, chemotherapy and radiotherapy^20^.

Several studies have been conducted to evaluate the effect of metformin or statins in combination with EGFR-TKIs on the prognosis of NSCLC patients. A small Chinese retrospective cohort study^21^, involving 90 patients with advanced NSCLC and EGFR-activating mutations, found that patients who received EGFR-TKI plus metformin were linked to prolonged PFS and OS than those treated with EGFR-TKI monotherapy. However, that study was limited by a small sample size and the retrospective design. In the present study, the combination of metformin with EGFR-TKIs did not prolong PFS and OS after adjusting for all covariates.

The combination of gefitinib with statins was shown to improve the response rate and PFS in previously treated patients with wild-type EGFR nonadenocarcinomas^22^. In another study, EGFR-TKIs (erlotinib or gefitinib) plus statins (atorvastatin or simvastatin) prolonged PFS in patients with advanced NSCLC harboring *KRAS* mutations^23^. However, the effect of a regimen combining statins plus EGFR-TKIs in patients with advanced NSCLC and activating EGFR mutations remains unclear. In a retrospective, nationwide, longitudinal cohort study^24^, administration of statin was associated with prolonged PFS and OS in patients with lung cancer receiving treatment with EGFR-TKIs. However, data on the pathology, AJCC TNM stage of lung cancer, BM, EGFR mutation status, and PS were not available in that study. In contrast, in the present study, these data were available. After adjustment for all factors, use of statins was not associated with prolonged TTF and OS in advanced NSCLC patients with EGFR-activating mutations who received first-line therapy with EGFR-TKIs. However, only 4.7% and 4.5% of the enrolled patients used metformin and statins, respectively. The detection power of this study may not be sufficient to detect the beneficial effects of metformin and statins.

EGFR-TKIs show physiological pH-dependent solubility, which may be susceptible to drug–drug interactions in the presence of antacids^25^. A retrospective population-based study^26^ demonstrated a possible negative clinical effect stemming from the co-administration of erlotinib and antacids. Of note, in that study^26^, patients were unselected for activating EGFR mutations. Two retrospective cohort studies^11,27^, involving 157 and 130 patients treated with erlotinib or gefitinib, respectively, for the treatment of EGFR mutation-positive advanced NSCLC have been conducted. These studies showed that the combination of EGFR-TKIs with antacid did not affect the efficacy of erlotinib and gefitinib. Our study corroborated this finding i.e., treatment with antacids did not alter the effectiveness of EGFR-TKIs on PFS and OS.

The use of glucocorticoids in the treatment of NSCLC remains controversial. The concomitant administration of glucocorticoids may reduce the efficacy of chemotherapy in patients with lung cancer^12^. A systematic review of randomized controlled trials involving glucocorticoid therapy indicated that patients with lung cancer receiving concomitant use of chemotherapy and glucocorticoids had worse outcomes than those who received chemotherapy alone^28^. However, the effect of glucocorticoids on the efficacy of EGFR-TKIs in patients with advanced lung cancer and EGFR-activating mutations remains unclear. In this study, we found that the concomitant use of glucocorticoids may reduce the efficacy of EGFR-TKIs in such patients. The results of the subgroup analyses were consistent with this finding. Other possible explanations for the higher risk of treatment failure and mortality associated with the use of glucocorticoids may be related to disease severity. In addition, excessive use of glucocorticoids has been associated with poor control of obstructive pneumonitis and BM, which may be the cause of this increased risk. Regardless of the causal nature of this association, chronic use of glucocorticoids is a powerful marker of high mortality risk in patients with lung cancer. Therefore, such patients warrant special attention.

The present study had several limitations. Firstly, we were unable to ascertain information on adverse events, which were not consistently recorded in administrative claims data. Secondly, our study used a retrospective design from the claims database of a tertiary referral hospital in Taiwan. Prospective studies are necessary to validate the present findings. Thirdly, we must acknowledge the lack of information on certain confounders (i.e., patient compliance, nutritional status, and body weight). The reasons for co-medication prescriptions are usually not explicitly documented in the patient record. Additionally, patients who expired or switched to other treatment due to disease progression within 3 months were excluded in this study. The results of our study might not reflect the real-world efficacy of three EGFR-TKIs as first-line treatment in patients with advanced NSCLC harboring EGFR mutations. Finally, the generalizability of our findings may be questionable, considering that almost all patients included in this study were Taiwanese. Future studies are warranted to verify these findings in non-Asian populations.

## Conclusion

This study demonstrated that administration of afatinib was associated with a lower risk of treatment failure compared to that observed following the administration of erlotinib and gefitinib in patients with advanced NSCLC harboring EGFR mutations. Moreover, the use of afatinib and erlotinib was associated with a lower risk of all-cause mortality compared to that observed following the use of gefitinib. The concurrent use of systemic glucocorticoids with EGFR-TKIs was associated with a higher risk of treatment failure and mortality. Further well-designed prospective studies, focusing on distinguishing the effects of different EGFR-TKIs in patients with advanced NSCLC harboring EGFR mutations, are warranted.

## Supplementary information

Supplementary Information.
