# Supplementary material for: The efficacy of first-line tyrosine kinase inhibitors combined with co-medications in Asian patients with EGFR mutation non-small cell lung cancer
Source: Sci Rep. 2020 Sep 11;10:14965. doi: 10.1038/s41598-020-71583-w (PMC7486374; doi:10.1038/s41598-020-71583-w)
Supplement: Supplementary file 1 — Supplementary Information. [file 41598_2020_71583_MOESM1_ESM.docx]

**The Efficacy of First-Line Tyrosine Kinase Inhibitors Combined with Co-medications in Asian Patients with Epidermal Growth Factor Receptor Mutation Non-Small Cell Lung Cancer**

Vincent Yi-Fong Su^1,5^, Kuang-Yao Yang^3,5,9^, Ting-Yu Huang^4^, Chia-Chen Hsu^2,8^, Yuh-Min Chen^3,5,7^, Jiin-Cherng Yen^4^, Yueh-Ching Chou^2,4,6,8*^ , Yuh-Lih Chang^2,4,8*^, Chien-Hui He^3^

^1^Department of Internal Medicine, Taipei City Hospital, Taipei, Taiwan

^2^Department of Pharmacy, Taipei Veterans General Hospital, Taipei, Taiwan

^3^Department of Chest Medicine, Taipei Veterans General Hospital, Taipei, Taiwan

^4^Department and Institute of Pharmacology National Yang-Ming University, Taipei, Taiwan

^5^Faculty of Medicine, School of Medicine, National Yang-Ming University, Taipei, Taiwan

^6^School of Pharmacy, Taipei Medical University, Taipei, Taiwan

^7^School of Medicine, Taipei Medical University, Taipei, Taiwan

^8^Faculty of Pharmacy, National Yang-Ming University, Taipei, Taiwan

^9^Cancer Progression Research Center, National Yang-Ming University, Taipei, Taiwan

These authors contributed equally: Vincent Yi-Fong Su and Kuang-Yao Yang

*Corresponding author, E-mail Address: ycchou@vghtpe.gov.tw, ylchang@vghtpe.gov.tw


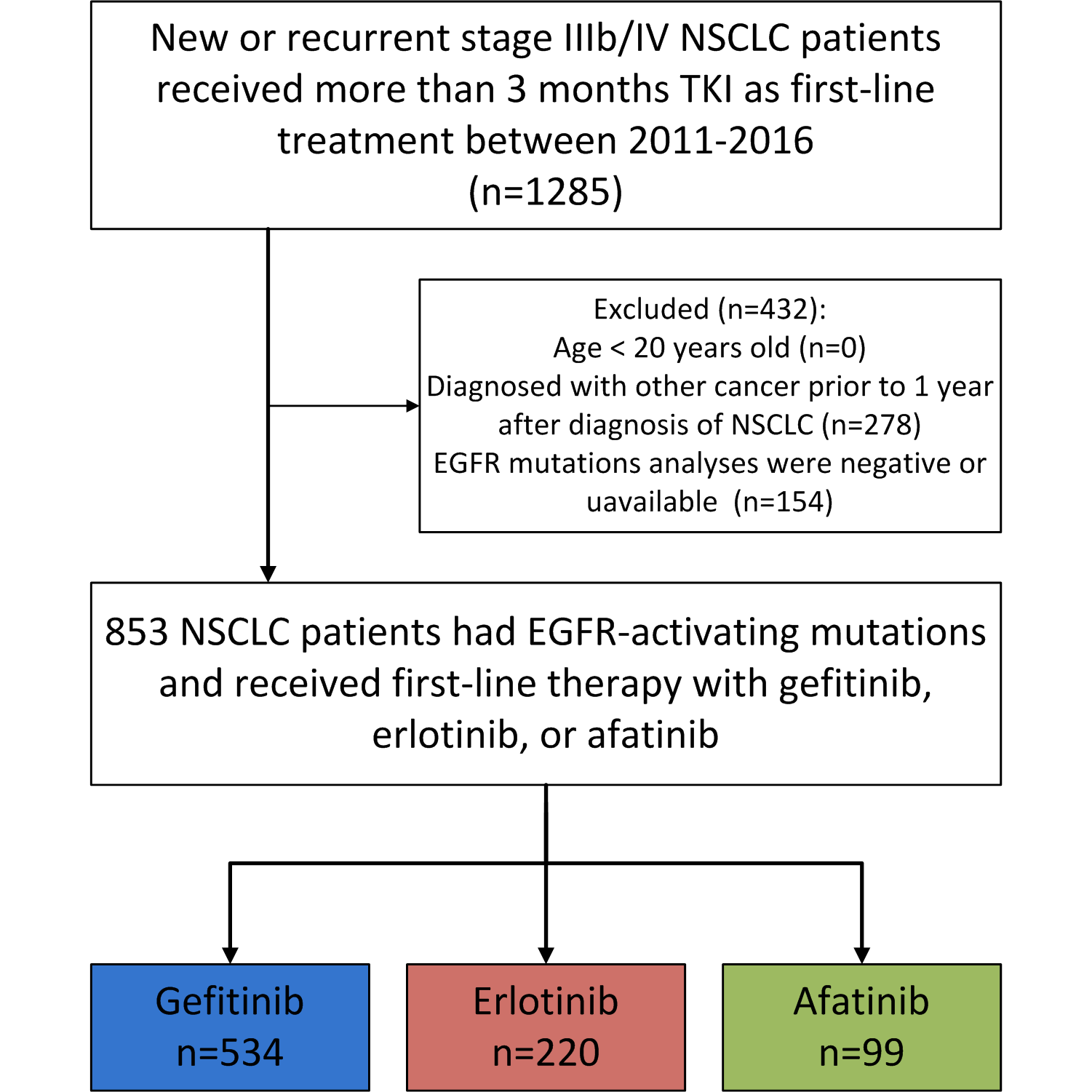


**Supplemental Figure 1. Flow diagram summarizing the process of enrollment**

**Supplemental Table 1. Baseline characteristics of the patient population (n=853)**

|  | Gefitinib (n=534) | | Erlotinib  (n=220) | | Afatinib  (n=99) | | Total  (n=853) | | P-value^#^ | |
| --- | --- | --- | --- | --- | --- | --- | --- | --- | --- | --- |
| Age, years (mean; SD) | 66.8 (12.8) | | 63.0 (12.4) | | 64.1 (10.8) | | 65.5 (12.6) | | 0.0004 | |
| Sex, (n; %) |  | |  | |  | |  | | 0.0043 | |
| Male | 173 (32.4) | | 90 (40.9) | | 47 (47.5) | | 310 (36.3) | |  | |
| Female | 361 (67.6) | | 130 (59.1) | | 52 (52.5) | | 543 (63.7) | |  | |
| Smoking (n; %) |  | |  | |  | |  | | <.0001 | |
| Never | 443 (83.0) | | 153 (69.5) | | 64 (64.7) | | 660 (77.4) | |  | |
| Current or ever | 91 (17.0) | | 67 (30.5) | | 35 (35.3) | | 193 (22.6) | |  | |
| Clinical stage (n; %) |  | |  | |  | |  | | 0.9710 | |
| IIIB | 20 (3.8) | | 9 (4.1) | | 4 (4.0) | | 33 (3.9) | |  | |
| IV | 514 (96.2) | | 211 (95.9) | | 95 (96.0) | | 820 (96.1) | |  | |
| EGFR mutation (n; %) |  | |  | |  | |  | | <.0001 | |
| Exon 19 deletion | 238 (44.6) | | 86 (39.1) | | 53 (53.5) | | 377 (44.2) | |  | |
| L858R | 272 (50.9) | | 129 (58.6) | | 31 (31.9) | | 432 (50.6) | |  | |
| Other | 24 (4.5) | | 5 (2.3) | | 15 (15.2) | | 44 (5.2) | |  | |
| Baseline brain metastases (n; %) |  | |  | |  | |  | | <.0001 | |
| Absence | 360 (67.4) | | 97 (44.1) | | 63 (63.6) | | 521 (61.1) | |  | |
| Presence | 174 (32.6) | | 123 (55.9) | | 36 (36.4) | | 332 (38.9) | |  | |
| ECOG PS (n; %) |  | |  | |  | |  | | 0.0017 | |
| 0&1 | 427 (80.0) | | 196 (89.1) | | 89 (89.9) | | 712 (83.5) | |  | |
| ≥2 | 107 (20.0) | | 24 (10.9) | | 10 (10.1) | | 141 (16.5) | |  | |
| Initial radiotherapy (n; %) |  | |  | |  | |  | | 0.6196 | |
| Yes | 90 (16.9) | | 38 (17.3) | | 13 (13.1) | | 141 (16.5) | |  | |
| No | 444 (83.1) | | 182 (82.7) | | 86 (86.9) | | 712 (83.5) | |  | |
| Deyo’s CCI (n; %) |  | |  | |  | |  | | 0.8354 | |
| 2 | 320 (59.9) | | 137 (62.3) | | 60 (60.6) | | 517 (60.6) | |  | |
| >2 | 214 (40.1) | | 83 (37.7) | | 39 (39.4) | | 336 (39.4) | |  | |
| Histology (n; %) |  | |  | |  | |  | | 0.1523 | |
| Adenocarcinoma | 505 (94.6) | | 202 (91.8) | | 96 (97.0) | | 803 (94.1) | |  | |
| Other | 29 (5.4) | | 18 (8.2) | | 3 (3.0) | | 50 (5.9) | |  | |
| Co-medications |  |  | |  | |  | |  | |  |
| Metformin (n; %)* | 27 (5.1) | 9 (4.1) | | 4 (4.0) | | 40 (4.7) | | 0.8064 | |  |
| Statins (n; %)* | 27 (5.1) | 10 (4.6) | | 1 (1.0) | | 38 (4.5) | | 0.2001 | |  |
| Antacids (n; %)* | 69 (12.9) | 15 (6.8) | | 8 (8.1) | | 92 (10.8) | | 0.0320 | |  |
| Glucocorticoids (n; %)* | 28 (5.2) | 21 (9.6) | | 4 (4.0) | | 53 (6.2) | | 0.0535 | |  |

*^#^p* values comparing baseline characteristics of the three TKI groups.

*Patients with co-medications were defined as those who had received co-medications (>28 cumulative defined daily doses) within first 3 months of TKI treatment initiation.

**Supplemental Table 2. Risk factor analysis of new brain metastases in patients with stage IIIB or IV NSCLC harboring EGFR-activating mutations**

|  | Univariate |  | Multivariate |  |
| --- | --- | --- | --- | --- |
|  | HR (95% CI) | P-value | HR (95% CI) | P-value |
| Age>=65 | 0.60 (0.31-1.13) | 0.113 | 0.56 (0.28-1.12) | 0.101 |
| Female | 0.57 (0.30-1.08) | 0.083 | 0.62 (0.30-1.28) | 0.194 |
| TKIs (Ref: Gefitinib) |  |  |  |  |
| Erlotinib | 1.67 (0.80-3.47) | 0.173 | 1.40 (0.64-3.05) | 0.398 |
| Afatinib | 0.59 (0.18-1.94) | 0.380 | 0.43 (0.12-1.49) | 0.181 |
| TKIs (Ref: Erlotinib) |  |  |  |  |
| Gefitinib | 0.60 (0.29-1.25) | 0.173 | 0.72 (0.33-1.56) | 0.398 |
| Afatinib | 0.35 (0.10-1.28) | 0.112 | 0.31 (0.08-1.20) | 0.090 |
| EGFR mutation (ref=Ex19d) |  |  |  |  |
| L858R | 1.49 (0.73-3.02) | 0.273 | 1.48 (0.71-3.12) | 0.299 |
| Other | 4.26 (1.50-12.1) | 0.007* | 7.48 (2.43-23.0) | <.001 |
| Stage IIIB (ref=IV) | 0.92 (0.22-3.81) | 0.904 | 0.89 (0.21-3.89) | 0.880 |
| ECOG PS≥2 | 0.67 (0.24-1.88) | 0.442 | 0.91 (0.31-2.72) | 0.869 |
| Current or ever smoking | 1.77 (0.89-3.51) | 0.102 | 1.64 (0.74-3.61) | 0.223 |
| Receiving Radiotherapy | 0.61 (0.15-2.55) | 0.503 | 0.51 (0.12-2.20) | 0.368 |
| DCCI Score >2 (Ref: 2) | 0.98 (0.51-1.89) | 0.960 | 0.83 (0.40-1.71) | 0.611 |
| Co-medications |  |  |  |  |
| Metformin | 1.67 (0.51-5.44) | 0.394 | 1.66 (0.44-6.34) | 0.458 |
| Statins | 1.59 (0.49-5.17) | 0.442 | 1.95 (0.53-7.22) | 0.318 |
| Antacids | 0.75 (0.23-2.44) | 0.633 | 0.86 (0.24-3.15) | 0.824 |
| Glucocorticoids | 2.92 (0.70-12.2) | 0.141 | 2.89 (0.60-13.8) | 0.184 |

* All factors were included in the Cox multivariate analysis.

**Supplemental Table 3. Risk factor analyses of treatment failure using propensity score adjustments**

|  | Gefitinib & Erlotinib | |  | Gefitinib & Afatinib | |  | Erlotinib & Afatinib | |
| --- | --- | --- | --- | --- | --- | --- | --- | --- |
|  | aHR (95% CI)* | P-value |  | aHR (95% CI)* | P-value |  | aHR (95% CI)* | P-value |
| TKIs |  |  |  |  |  |  |  |  |
| Gefitinib | Ref |  |  | Ref |  |  | - |  |
| Erlotinib | 0.87 (0.73-1.05) | 0.157 |  | - |  |  | Ref |  |
| Afatinib | - |  |  | 0.56 (0.42-0.73) | <0.001 |  | 0.56 (0.41-0.77) | <0.001 |

* Propensity score and all factors were included in the Cox multivariate analysis.
